# Small Molecule Fluorescent Probes for G- Quadruplex Visualization as Potential Cancer Theranostic Agents

**DOI:** 10.3390/molecules24040752

**Published:** 2019-02-19

**Authors:** Pallavi Chilka, Nakshi Desai, Bhaskar Datta

**Affiliations:** 1 Department of Biological Engineering, Indian Institute of Technology Gandhinagar, Gandhinagar, Gujarat 382355, India; pallavi.chilka@iitgn.ac.in (P.C.); nakshi.desai@iitgn.ac.in (N.D.); 2 Department of Chemistry, Indian Institute of Technology Gandhinagar, Gandhinagar, Gujarat 382355, India

**Keywords:** G-quadruplexes, G-quadruplex probe, oncogene promoter, cancer theranostic, quadruplex visualization

## Abstract

G-quadruplexes have gained prominence over the past two decades for their role in gene regulation, control of anti-tumour activity and ageing. The physiological relevance and significance of these non-canonical structures in the context of cancer has been reviewed several times. Putative roles of G-quadruplexes in cancer prognosis and pathogenesis have spurred the search for small molecule ligands that are capable of binding and modulating the effect of such structures. On a related theme, small molecule fluorescent probes have emerged that are capable of selective recognition of G-quadruplex structures. These have opened up the possibility of direct visualization and tracking of such structures. In this review we outline recent developments on G-quadruplex specific small molecule fluorescent probes for visualizing G-quadruplexes. The molecules represent a variety of structural scaffolds, mechanism of quadruplex-recognition and fluorescence signal transduction. Quadruplex selectivity and in vivo imaging potential of these molecules places them uniquely as quadruplex-theranostic agents in the predominantly cancer therapeutic context of quadruplex-selective ligands.

## 1. G-Quadruplexes (G4) and Cancer

Cancer biology is inherently complex owing to the varied cellular alterations and mutations juxtaposed with failures of biological safety mechanisms. Molecular complexity of tumors adds to complexity of the disease, which is evident from the fact that no universal cancer biomarker is yet to be documented [1]. Identification of systemic and molecular targets unique to cancer cells has paved the way for development of effective anticancer agents with minimal impact on normal cells. Over the last two decades, G-quadruplex (G4) nucleic acids have emerged as an important component of cancer research [2]. Profound impact of G4 structures on transcriptional regulation, replication, telomere stability and chromatin remodeling have facilitated their consideration as potential targets for cancer therapeutics [3]. G4 DNA has been suggested as being deeply linked with replication and maintenance of genomic stability [4]. In particular, biological events relying on the presence of DNA in single stranded form could be influenced by formation of G-quadruplexes. G4 RNA has also been reported as having transcriptome-wide presence with possible roles in transcription regulation and chromatin organization [5,6]. G4 DNA have been purported as epigenetic factors that regulate expression of cancer related genes [7]. Putative G quadruplex motifs are overrepresented in proto-oncogenes by 69% as compared to tumor suppressor genes. Hence, they are perceived as attractive targets for anticancer agents [7,8]. G4-targeting ligands have been demonstrated to trigger cellular responses correlated with the perceived function of corresponding quadruplexes [9,10]. Nevertheless, distinguishing causality and effect of G4 structures in the context of cancer is challenging. Further, the influence of these structures on inception and progression of cancer, if any, is sparsely understood. This is due to challenges in detection and analysis of cellular responses that could be directly correlated to these structures in living cells. The ability to visualize or image G4 structures offers a frontier area of research that is likely to enhance our understanding of G4-associated physiological processes. The design of efficient G4-targeting anti-cancer drugs is likely to become more straight-forward with improved insight into the mechanisms and molecular pathways of G-quadruplexes. This review juxtaposes G4 visualizing agents within the larger set of G4-targeting ligands with a view towards development of potential theranostic agents.

### 1.1. G4 as Molecular Targets

The stabilization of G4 structures in non-telomeric regions such as proto-oncogenes and promoter regions has been shown to provide transcriptional control resulting in anti-proliferative and anti-tumor activities in several in vitro and in vivo models of human cancers [11,12]. The stabilization of G4 structures in telomeres by small molecules inhibits telomerase activity and induces apoptosis in cancer cells [13]. Small molecules selectively targeting and stabilizing G4 structures are inextricably linked with the development of new anticancer drugs. G4 RNAs have also been suggested as possessing therapeutic potential [14]. G4-structures adopted by DNA vary based on numerous factors including local ion concentration of cations that can stabilize the anionic G4 tetrads. G4 structures in proto-oncogenes are stabilized at physiologically relevant K^+^ concentrations such as 100 mM thereby attenuating transcription. Among other changes in aggressive tumors, over-expression of K^+^ membrane ion channels leads to depletion in intracellular K^+^ concentration to nearly 60 mM [15]. In vitro studies have shown that while there are no G4 structural changes at these K^+^ concentrations, their overall stability is reduced. Thus, destabilization of G4 structures and cancer progression are synergistically connected [15]. During tumor progression, the transcriptional control of proto-oncogenes is removed. Nevertheless, loss of such control is a complex event, the presence of G-rich sites and concomitant possibility of G4 structures acting as contributing factors is yet to be clearly proven. Identification of molecular level impact of G4 structures on vital processes associated with cancer is likely to open new avenues in cancer therapy and diagnostics. The interplay of two specific areas is important in this regard: (i) G4 structures formed in cancer cells that can be potential molecular targets and (ii) small molecules with ability to bind, stabilize and possibly visualize G4 structures.

### 1.2. G4 Stabilizing Agents for Cancer Therapeutics 

Putative G-quadruplex-forming sequences are located throughout the genome in a non-random fashion. They have been found to be mainly concentrated in telomeric regions and promoters and UTRs of genes associated with cell proliferation and cancer [16]. Molecular targets from the plethora of sites across genome are discrete for every cancer type depending upon the genes over-expressed. Nevertheless, the most prominently G4-associated genes in-clude MYC, KIT, BCL2, VEGF, and KRAS. 

**Figure 1:**
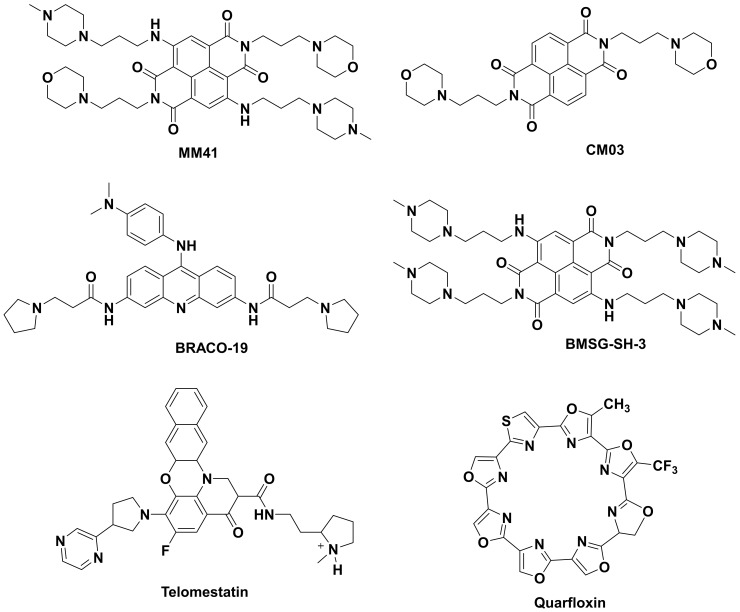
Structures of G4-stabilizing ligands.

One of the most difficult cancers to treat is human pancreatic ductal adenocarcinomas (PDAC) which is predominantly (>95%) directed by oncogenic KRAS [17]. Apart from KRAS, BCL2 gene is also over-expressed in pancreatic cancer [18]. The naphthalenediimide derivative MM41 (Figure 1) is a ligand reported to down-regulate transcription of BCL2 and KRAS genes by stabilizing G4 structure formed in their promoter with higher affinity for BCL2 gene [19]. CM03 (Figure 1) is a better and more effective derivative of MM41 which harbors higher levels of tolerability and targets other putative quadruplex containing genes such as PAK1, ROBO3, and PLXNA1. All of these are highly over-expressed in PDAC. Animal studies have shown that CM03 effectively stops tumor growth and the antitumor activity continues even after the dose is ceased. The action of CM03 has been attributed to the down-regulation of genes involved in PDAC survival and metastasis via G4 stabilization [20,21].

The most common mechanism exploited by cancer cells to achieve uncontrollable growth is over-expression of telomerase noted by up-regulation of hTERT. In uterus carcinoma cell line and glioblastoma cell lines, a decrease in hTERT expression leads to cellular senescence, growth arrest, decrease in tumor size and preferential apoptosis of Glioma stem cell (GSC) [19,22]. A tri-substituted acridine ligand BRACO-19 (Figure 1) inhibits telomerase activity by drastically reducing hTERT expression resulting in telomere shortening and end-to-end chromosomal fusions in cancer cells as a consequence of G4 stabilization at the ssDNA overhang. Apart from BRACO-19, BMSG-SH-3 and telomestatin (Figure 1) are other telomere G4 stabilizers that have been reported to cause cell cycle arrest and apoptosis in cancers of uterus, glioblastoma, prostate and gastrointestinal tissues. Quarfloxin, a fluoroquinolone derivative with anti-neoplastic activity [23], is to date the only G4 ligand that has reached Phase II clinical trials although it was eventually withdrawn due to bioavailability-related issues [24]. Research into G4-targeting ligands has been from a predominantly therapeutic perspective. NMR studies on the interaction of G-quadruplexes with specific ligands in living cells of X. laevis oocytes have suggested that ligand binding to the quadruplex oligonucleotide promotes binding of other biomolecules [25]. Such changes in quadruplex structure upon ligand binding hamper analysis of quadruplexes by NMR. Thus, more advanced techniques which can highlight real time interaction of ligand with G-quadruplexes are required. In the following sections, we review G4-targeting ligands that enable visualization of the secondary structures. The quest for an ideal probe with extraordinary G4 selectivity and fluorescence enhancement has yielded several ligands with exclusive properties. The oldest known synthetic dyes, namely cyanine derivatives, have been used to probe G-quadruplexes. In this regard, thiazole orange which was already known as a prominent nucleic acid staining agent was shown to display high affinity for the cationic quartets of G-quadruplexes. Other G4-selective fluorescence probes have been built around pyridinium, carbazole and benzothiazole moieties, albeit in a semi-empirical manner. The interplay of probe structure and quadruplex topology has been reviewed previously, especially with respect to implications on binding affinity and enhancement of fluorescence [26]. These molecules are potent G4-DNA stabilizing agents and also qualify for a probe to detect G4-DNA. First among these possible theranostic agents is BMVC, a carbazole diiodide derivative which suppressed tumor progression in H1299 cancer cells, primarily by accelerating telomere shortening which is a result of G-quadruplex stabilization in telomeres. It also had some off-target effects, all of which resulted in suppression of cancer cell migration. BMVC is described in detail later in this article as a small molecule fluorescent probe for quadruplex visualization [27]. Ligands bearing such therapeutic and diagnostic dual property are still limited because a small molecule should have least cytotoxicity to qualify as a fluorescence probe that is suitable for live cell imaging.

### 1.3. Visualizing G4 Structures in Cellular Context

The pivotal role of G4 structures in manifestation and progression of cancer has been already been discussed in this review. The understanding of G4 distribution in normal versus cancerous cells would foster development of better tools that can selectively target DNA or RNA G-quadruplex at any given cell cycle phase [28]. The visualization of DNA and RNA G4 structures can be accomplished by immune-detection, in situ fluorescence labeling of G4 ligands and quadruplex selective small molecular fluorescence probes. These approaches enable study of the selectivity of ligands for DNA or RNA G4 under defined cellular conditions [29]. The transient nature of G4 structures and their resolution in the genome and transcriptome makes it challenging to study the formation of G4 in eukaryotic cells. Visual tools can aid to assess G4 formation during metabolic processes as well as during cellular differentiation [30]. BG4 antibody has provided an opportunity to probe the cellular role of G-quadruplexes and also their potential involvement in disease. Biffi et al., have probed nuclei of human tissues with BG4 antibody to detect formation of DNA G-quadruplex in them. They successfully quantified BG4 positive cell nuclei in patient stomach and liver cancers and also non-neoplastic tissues [29]. Their results indicated significantly higher percentage of BF4 foci in cancerous tissue as compared to non-neoplastic tissues which indicates that processing of G-quadruplex is mis-regulated in some human cancers. This could be a result of alterations in cellular processes that regulate genome stability or changes in the chromatin state at G-quadruplex sites in cancer tissues [31]. 

**Figure 2:**
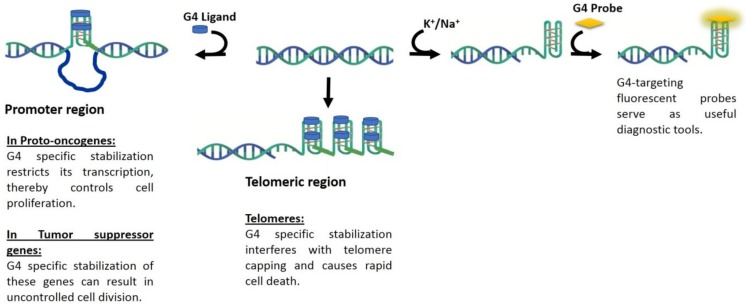
Schematic representing action of G4-stabilizing ligands and G4-targeting fluorescent probes.

In the following section we review some of the promising fluorescence probes that have been reported for G4 visualization. Agents that are capable of G4-visualization may also be considered as potential theranostic agents, as represented schematically in Figure 2.

## 2. Fluorescence Probes for G4 Visualization

### 2.1. BMVC

**Figure 3:**
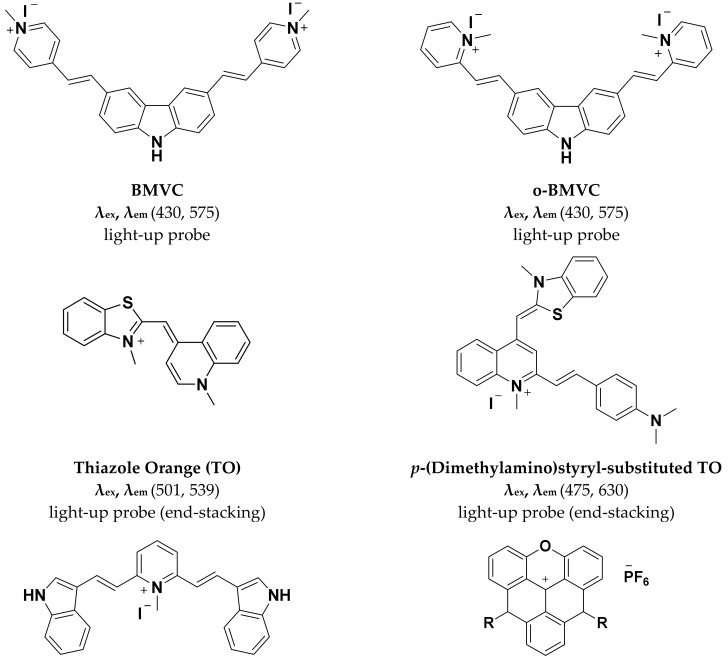

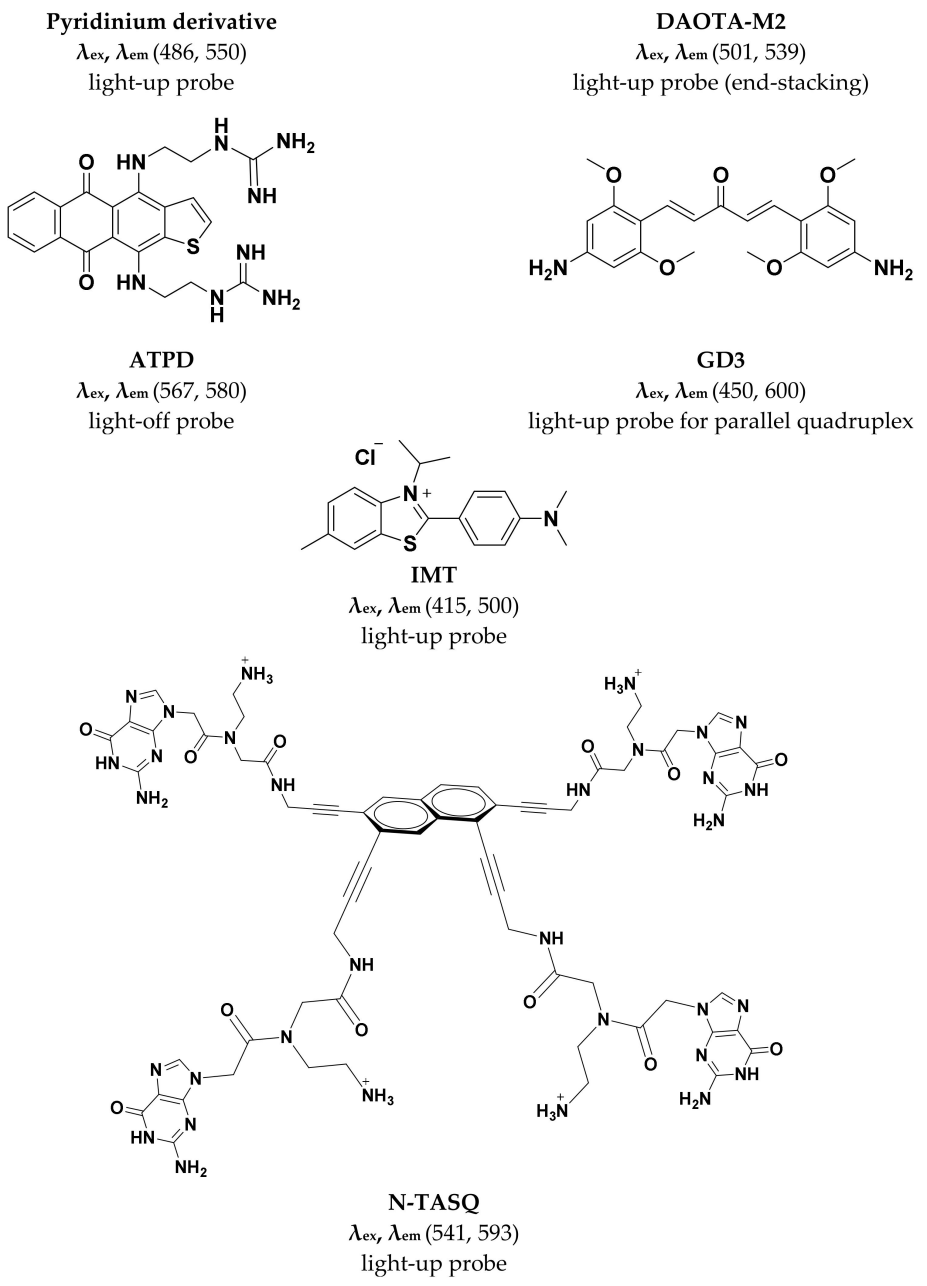
Small molecule G4-selective fluorescent probes reviewed in this article.

The molecule 3,6-bis(1-methyl-4-vinylpyridinium)carbazolediiodide (BMVC, Figure 3) was first reported by Chang and coworkers for its ability to recognize the unimolecular G-quadruplex structure formed by the human telomeric sequence d(T2AG3)4 [32]. This telomeric sequence named Hum24 is known to fold into distinctly different structures in presence of Na+ versus K^+^ [33,34]. BMVC exhibits low cytotoxicity and is capable of inhibiting telomerase activity at low concentrations (~50 nM).

The carbazole derivative was subsequently used for spatial imaging of telomeric antiparallel quadruplexes. Two photon excitation fluorescence lifetime imaging microscopy was used to estimate the differences in fluorescence decay times of BMVC upon interaction with various nucleic acid structures. The interaction of BMVC with the parallel and antiparallel forms of Hum24 exemplifies an important challenge towards selective quadruplex staining, namely distinguishing between different quadruplex topologies. A difference in the fluorescence lifetime of BMVC was used to distinguish parallel from antiparallel quadruplex structures formed by d(T2AG3)4. Lifetimes of BMVC in the range of 1.85–2.2 ns were considered as indicative of interaction with antiparallel quadruplexes while lifetimes are in the range of 1.2–1.85 ns denoting interaction with other nucleic acid structures. This property was also used for staining metaphase chromosomes in nasopharyngeal carcinoma KJ-1 cells and mapping the localization of antiparallel quadruplexes therein. The antiparallel quadruplexes are identified in regions that are mostly at the ends of the chromosomes. BMVC has been suggested as mostly binding to the basket form of telomeric quadruplexes stabilized by Na+ by external π-stacking. While the binding preference of BMVC for Hum24 quadruplex was clearly superior compared to the cognate duplexes, its ability to distinguish antiparallel from parallel telomeric quadruplexes remains ambiguous. This has been partly attributed to the polymorphism of telomeric quadruplexes in presence of K^+^ as well as the greater amount of K^+^ compared to Na^+^ inside cells. Nevertheless, BMVC was useful for identifying specific zones on chromosomes where the antiparallel quadruplexes are localized. BMVC is found to selectively light up cancer cells compared to normal cells. This has been attributed to the ability of the molecule to escape from lysosomal retention in cancer cells, leading to association with the mitochondria and nucleus with subsequent fluorescence enhancement [35]. The ability of BMVC to reveal cellular behaviour during carcinogenic transformation has been studied, though these studies do not invoke quadruplexes in their underlying hypothesis [36]. The singular behaviour of BMVC in cancer cells has helped in the development of a remarkable application for identifying pre-cancerous lesions. The application in the form of a handheld device is promising as a sensitive method for routine point-of-care screening of cancerous cells and early cancer detection. The behaviour of BMVC in cancerous cells has also led to its use in proving the existence of mitochondrial quadruplex DNA [37]. In spite of BMVC exhibiting attractive selectivity for G-quadruplex structures, its wider use is hampered by the relatively low wavelength of excitation of the molecule at –430 nm. Further, the absorbance spectrum of BMVC overlaps in the UV-region where DNA, proteins and tissues exhibit indigenous absorbance and fluorescence. Nevertheless, BMVC has emerged as one of the most important contenders for selective staining and identification of quadruplex structures in vivo. 

### 2.2. Pyridinium Derivatives

G-quadruplexes are notable for the diversity in their folding topologies and resulting structural symmetry. The correlation between structural symmetry underlying G-quadruplexes and quadruplex-binding probes has been investigated through suitably modified pyridinium derivatives [38]. Alteration in the side group modification of pyridinium conjugates was used to obtain asymmetric (C1 symmetric) and symmetric (C2 and C3 symmetric) dyes. The styrene-like substitutions on pyridinium in these conjugates can rotate freely resulting in weak fluorescence. Interaction of the conjugates with quadruplex DNA restricts their rotation thereby leading to fluorescence enhancement. 

**Figure 4:**
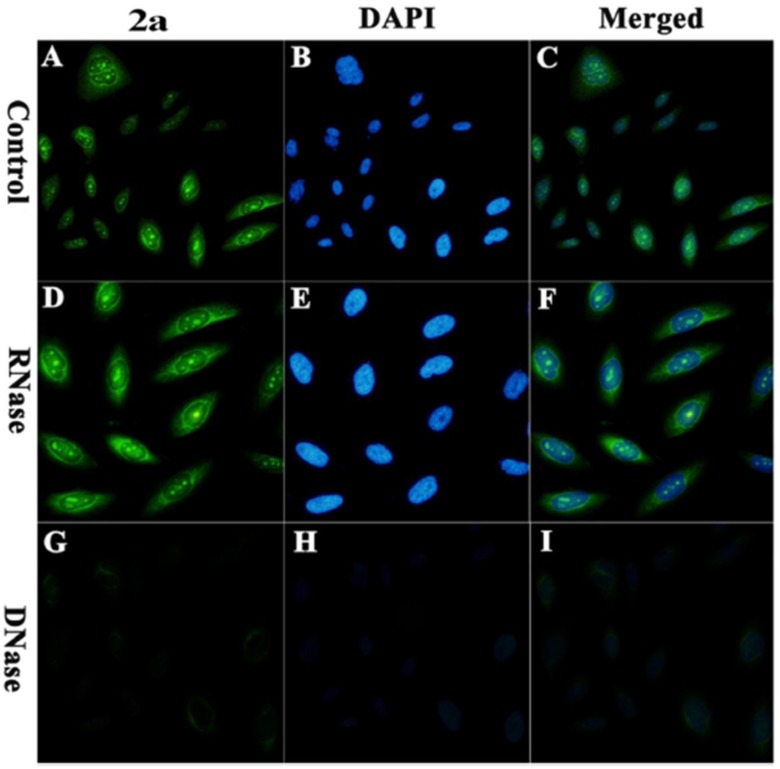
Fluorescence microscopy of live PC3 cells stained with pyridinium derivative and DAPI. The fluorescent foci corresponding to probe observed in merged image is speculated to be rDNA rich nucleoli which is G rich and has potential to fold into G4 structures. (adapted with permission from [38]).

Several derivatives were synthesized based on the above principles and based on performance in live cell imaging suitable molecules were chosen for further study. The indolyl-substituted pyridinium compound, 2,6-bis-((*E*)-2-(1*H*-indol-3-yl)-vinyl)-1-methylpyridin-1-iumiodide) (Figure 3), bearing C2 symmetry was found to display superior selectivity for quadruplex DNA compared to the other derivatives with nearly 50-fold enhancement in fluorescence in presence of the human telomeric sequence telo21. The ability of indolyl-substituted pyridinium to effectively target quadruplex DNA was evident from their high equilibrium binding constant and low limit of detection (33 nM) for telo21. The molecular symmetry of the indolyl-substituted pyridinium possibly aligns well with the four-fold symmetry of quadruplex DNA. The pyridinium derivative displays interesting nuclear staining characteristics (Figure 4). Staining of live pancreatic cancer (PC3) cells showed strong fluorescence response in the nucleoli and is attributed to guanine-rich rDNA forming temporal quadruplex conformations. The pyridinium conjugates are similar in live cell staining behavior to styryl-modified TO dyes in their resistance to RNase but not DNase pre-treatment. Cell cycle progression in live PC3 cells was visualized using the indolyl-substituted pyridinium dye [38]. A substantial enhancement in fluorescence was observed at the G1/S phase checkpoint in contrast to weak fluorescence at the G0/G1 phase. The remarkable fluorescence response of the dye at the G1/S phase is attributed to increase in replication-dependent quadruplex formation. While the cell staining application of pyridinium derivatives is promising with respect to identification of putative quadruplex structures in vivo they also show a propensity to bind other regions of the cell. Further research on this aspect will facilitate wider application of the pyridinium conjugates.

### 2.3. Thiazole Orange and Analogues

In contrast to the relatively recent discovery of BMVC, thiazole orange (TO) is one of oldest known cyanine dyes and has been one of the most widely used duplex nucleic acid staining reagents [39,40]. Notably, binding of TO with duplex DNA is reversible and can dissociate during gel electrophoresis [41]. This aspect was addressed by the development of dimeric TO dye TOTO which displayed significantly greater affinity for duplex DNA compared to the monomeric counterpart. Interestingly, TO has been shown to bind very strongly to triplex and quadruplex DNA followed by nearly 1000 fold fluorescence enhancement [42]. 

**Figure 5:**
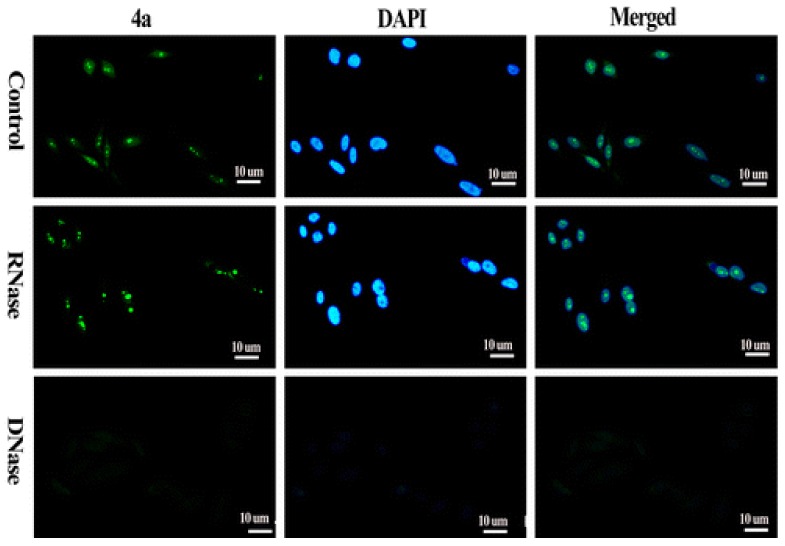
The compound 4a is p-(dimethylamino)styryl substituted TO. Fluorescence micros-copy of PC3 cells shows that the probe 4a enters the nucleus and foci correspond to rDNA of nucleoli that is guanine rich and can assume G4 conformations. The dramatic decrease in fluorescence intensity is suggestive that probe has a preference for DNA secondary structure. (Adapted with permission from [43]).

Comparative staining of osteosarcoma U2OS cells with TO and DAPI revealed the preference of TO for certain areas of the nucleus in contrast to uniform staining of the entire nucleus by DAPI. Interestingly, TO-quadruplex complexes were stable and did not dissociate during size exclusion chromatography. The mode of TO binding to quadruplexes has been suggested as intercalative based on the increase in length of the DNA molecule upon dye binding. The high fluorescence quantum yield of TO upon G-quadruplex binding has spurred the development of TO derivatives that are capable of more selective G-quadruplex recognition. The integration of G-quadruplex specific binders into the TO molecule is one such approach that has been explored. Pyridodicarboxamide (PDC) bisquinolinium conjugated to TO displayed nearly 8-fold improvement in fluorescence enhancement upon interaction with quadruplex targets compared to unmodified TO [44]. Similarly, the introduction of a benzofuroquinolinium moiety on the TO scaffold has permitted superior and selective recognition of quadruplexes formed by c-myc oncogene promoter and human telomeric sequence (telo21) [45]. A fluorescence enhancement in the vicinity of 300-fold is observed upon interaction of the TO conjugate with c-myc and telo21. The signaling role in both of these TO derivatives is attributed to TO while the benzofuroquinolinium or PDC moiety is considered as the quadruplex-binding unit. Other examples of integrating G-quadruplex specific binders with TO include the 4-(dimethylamino) styryl (Figure 3) derivative that exhibit 10-fold greater selectivity for G-quadruplexes as opposed to duplex DNA [46]. Live cell staining of human pancreatic cancer (PC3) cells with the 4-(dimethylamino) styryl modified TO show the ability of the dye to stain the nucleoli (Figure 5). A high background fluorescence of the dye was attributed to non-specific interactions with duplex DNA as well as binding of the dye with RNAs in the nucleus. The enhanced fluorescence of dye in nucleoli was lost upon treatment of cells with DNase but not RNase. Interestingly, other styryl modified TOs have been used by the same group as RNA-selective fluorescent dyes [46]. 4-(Dimethylamino)styryl-modified TO exhibits attractive cell tolerability (IC50 = 0.59 mM) and photostability. The success of TO alone and in conjunction with quadruplex-binders, towards selective staining of quadruplexes makes it a prominent candidate for practical applications and further refinement.

### 2.4. IMT

IMT, a benzothiazole derivative, displays extraordinary fluorescence selectivity for nearly all types of G4-DNA and G4-RNA structures over i-motif, duplex and single stranded nucleic acid targets [47]. A major disadvantage of using chemical probes for live cell imaging is off-target binding which substantially decreases signal to noise ratio. 

**Figure 6:**
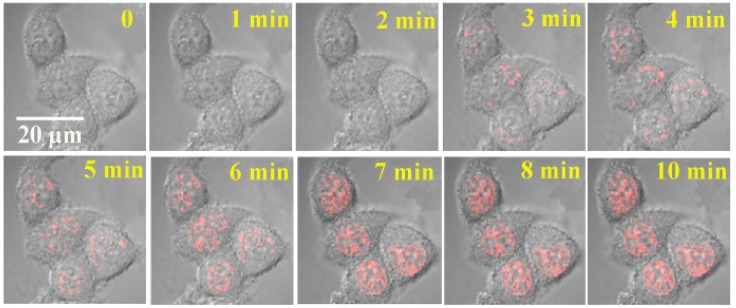
The fluorescent foci from G-quadruplex specific IMT probe (4µM) as seen in live HeLa cells with time lapse CLSM. (adapted with permission from [47]).

Tang and coworkers tested interaction of IMT with amino acids, vitamins, proteins, glucose and several metal ions which can possibly interfere with G-quadruplex staining in cellular conditions. A sharp contrast of fluorescence enhancement was observed for IMT with G-quadruplex over other components. Furthermore, CD and FRET analysis precluded any stabilizing effect of IMT on G-quadruplex structures. The researchers established the applicability of IMT for G-quadruplex visualization by fixed cell and live cell imaging techniques (Figure 6) [47]. It was observed that IMT co-localized with BG4 (G4 specific antibody) and was able to report increase in fluorescent foci during cell cycle progression from G0 to S phase. Till date, it is the best known probe which can trace real-time changes in G4 structures in living cells. Their results pave a way to understand biological response of G-quadruplexes to drug treatments and cellular consequences that alter upon modulating the stability of these structures.

### 2.5. N-TASQ

In a novel strategy towards the development of G-quadruplex targeting ligands, the quadruplex structural motif has been sought as a template for the assembly of synthetic quartets [48]. Signal transduction by the assembled quartets serve as an indication of quadruplex-recognition. This strategy and the corresponding class of compounds are called template assisted synthetic G-quartet or TASQ. TASQs have been suggested as twice-as-smart ligands and beginning with calixarene, several ligands such as pyrene and naphthalene have been examined for their ability to assemble synthetic G-quartets [49,50]. Among these naptho-TASQ or N-TASQ have been used for the direct visualization of RNA G-quadruplexes. N-TASQ like other TASQ agents works as a fluorescence light-up probe. The fluorescence of unbound N-TASQ is weak due to quenching via intramolecular electron transfer from surrounding guanines. 

**Figure 7:**
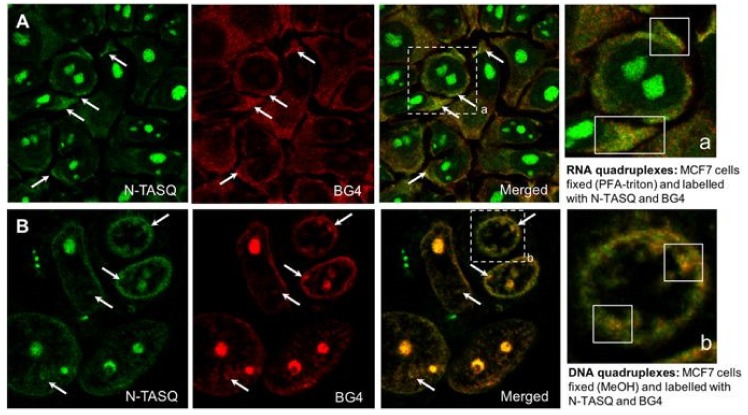
Confocal microscopy of MCF7 cells treated with N TASQ and BG4 (G-quadruplex specific antibody) and fixed with methanol indicates specificity of the probe for RNA and DNA G-quadruplex structures. (adapted with permission from [30]).

Interaction of N-TASQ with suitable quadruplexes disrupts the electron transfer from guanines thereby leading to significant fluorescence enhancement. Multi-photon microscopy has been used to visualize N-TASQ in human breast cancer (MCF7), human osteosarcoma (U2OS) and murine melanoma (B16F10, Figure 7). Staining of RNA quadruplexes is inferred from the persistent fluorescence contrast between cytoplasm and nucleus for RNase treated cells [50]. Nevertheless, RNase digestion is accompanied by a reduction in total fluorescence signal while the nucleoli remain weakly fluorescent. The possibility of interaction of N-TASQ with non-nuclear sub-cellular entities in addition to the near-UV absorbance displayed by the dye, restrict its scope of use. Interestingly, the ligand is compatible with confocal microscopy based on its uncommon spectroscopic property called Red Edge Effect (REE). N-TASQ in interaction with quadruplex-DNA displays a dependence of the wavelength of emission maximum on the excitation wavelength and can thus be visualized by lasers that are commonly associated with confocal microscopy. Fluorescence imaging of MCF7 cells demonstrated the presence of RNA quadruplexes in PFA-triton fixed cells and DNA quadruplexes in MeOH-fixed cells [30]. The images with low cytoplasmic fluorescence and strongly stained nucleoli were characterized as RNA quadruplexes whereas the DNA quadruplexes were represented by discrete nuclear fluorescent foci. The potential of N-TASQ in direct visualization of G-quadruplexes was further confirmed by co-labeling of MCF7 cells with quadruplex-specific antibody, BG4 and N-TASQ ligand. Laguerre et al. had observed BG4 labeled quadruplexes mostly in cytoplasmic sites (RNA quadruplexes) after PFA-triton fixation and in nuclear sites (DNA quadruplexes) after MeOH fixation [50]. Although fluorescence of BG4 supersedes N-TASQ, the merged fluorescence images suggested that both BG4 and N-TASQ have similar cellular targets in cytoplasm and nucleus [50]. N-TASQ represents an important variation in the paradigm of quadruplex-recognition and visualization in cells. While limitations of the probe in terms of sensitivity for other cellular targets are acknowledged, it has expanded the scope for discovery of quadruplex-specific red edge probes.

### 2.6. GD3

Pyridodicarboxamide derivatives have been successful G4 specific ligands with their applications as potential anti-cancer and anti-inflammatory drugs. However, its unexplored fluorescent probe based application was brought to light by Yang and fellow researchers by synthesizing and studying its derivative, tetramethoxybis(4-aminobenzylidene) acetone, GD3 (Figure 3) [51]. Their results demonstrated that GD3 has superior selectivity for parallel G-quadruplex topology and is weakly fluorescent in presence of ssDNA, dsDNA and triplexes. This derivative did not affect the folding of G4-DNA and therefore was investigated for intracellular G-quadruplex visualization in ARPE-19 cell lines [51]. The fixed cell staining images displayed bright red fluorescent foci in regions coinciding with nucleoli along with emission from cytoplasm. The nuclear fluorescence dramatically reduced upon DNase treatment but RNase treatment did not reduce the cytoplasmic fluorescence which suggests that off-target interactions of GD3 in cytoplasm. Thus, more research is necessary before bis(4-aminobenzylidene) acetone derivatives can be established as G4 specific cellular fluorescent probe. Nevertheless, this is a pioneering study with regards to development small molecular fluorescent probe that can distinguish different G-quadruplex topologies.

### 2.7. Other Agents

A few other probes have been reported in addition to the above prominent G-quadruplex visualizing agents. Notably, the triangulenium derivatives ADOTA-M and DAOTA-M2 (Figure 3) exhibit nucleic acid topology-dependent fluorescence lifetime analogous to BMVC [52]. Interaction of DAOTA-M2 with G-quadruplexes resulted in higher emissive lifetime of the probe compared to duplex and single stranded DNA. While FLIM was used for probing DAOTA-M2 in osteosarcoma (U2OS) cells, localization of the probe in quadruplex-forming regions remains inconclusive due to overlapping lifetimes obtained from interaction with RNA sequences versus quadruplex structures. Among other probes, binapthyl amines have been shown in vitro to possess high specificity and affinity for the quadruplex formed by c-Myc [53]. While the binapthyl amine dyes were able to stain HeLa cells and illustrate the down regulation of c-Myc, these were not indicative of their direct binding to G-quadruplexes. Apart from BMVC, another class of carbazole derivatives namely BPBC has been found to display attractive selectivity for parallel quadruplexes [54]. However, staining of human MCF-7 cells with BPBC shows very weak fluorescence response that is attributed to the dispersed localization of the dye in cytoplasm. Guanidino anthrathiophenediones (ATPDs) such as 4,11-bis[(ω-guanidinoethyl)amino]anthra[2,3-b]thiophene-5,10-dione (Figure 3) are yet another class of quadruplex-selective ligands that have been used for live cell visualization of quadruplexes [55]. While some guanidino ATPDs exhibit granular nuclear distribution, they are not localized only in the nucleus. Further, the potential of these compounds as imaging agents is somewhat interrupted by their ability to down-regulate the activity of the HRAS promoter. Nevertheless, the selective uptake of ATPDs by malignant T24 bladder cells over non-malignant kidney cells, in combination with their cellular localization makes them promising theranostic reagents.

## 3. Conclusions and Perspectives

Over the past decade, massive headway has been made towards the development of G4-selective ligands that are capable of stabilizing the secondary structures. The quest for quadruplex-specific ligands has navigated the challenge of distinguishing quadruplexes from canonical DNA in the early days, to the challenge of distinguishing quadruplex topologies from one another at present time. Not surprisingly, fluorescent ligands capable of identifying quadruplex structures in vivo have faced similar challenges. Several scaffolds such as carbazoles and pyridinium derivatives have emerged as promising building blocks for construction of quadruplex-selective staining agents. G4-selective fluorescence probes most commonly rely on an end-stacking mechanism of recognition. Recurring motifs in these probes include heterocyclic moieties such as benzothiazole, presence of charged centers and extended conjugation. Considering the much smaller set of G4-selective fluorescent ligands, it is conceivable that such agents may fulfill a dual theranostic role enabling wider validation of G4-targets in disease contexts (Figure 2). Current challenges facing the development of such quadruplex-selective theranostic agents include unambiguous intracellular localization and ability to report functional aspects of quadruplexes.

## Abbreviations of G4 Selective Fluorescence Probes

BMVC3,6-bis(1-methyl-4-vinylpyridinium)carbazolediiodideTOThiazole OrangeIMTN-Isopropyl-2-(4-N,N-dimethylanilino)-6-methylbenzothiazoleN-TASQNaptho-Template Assisted Synthetic G-QuartetGD3Tetra-methoxy bis(4-aminobenzylidene) acetoneDAOTA-M2Morpholino containing bis-substituted trianguleniumADOTA-MMorpholino containing mono-substituted trianguleniumBPBC9-methyl-3,6-bis[5-(4-methylpiperazin-1-yl)-1Hbenzo[d]imidazol-2-yl]-9H-carbazole ATPDsGuanidino anthrathiophenediones
